# Selection of Reliable Biomarkers from PCR Array Analyses Using Relative Distance Computational Model: Methodology and Proof-of-Concept Study

**DOI:** 10.1371/journal.pone.0083954

**Published:** 2013-12-12

**Authors:** Chunsheng Liu, Hongyan Xu, Siew Hong Lam, Zhiyuan Gong

**Affiliations:** Department of Biological Sciences, National University of Singapore, Singapore, Singapore; Memorial Sloan Kettering Cancer Center, United States of America

## Abstract

It is increasingly evident about the difficulty to monitor chemical exposure through biomarkers as almost all the biomarkers so far proposed are not specific for any individual chemical. In this proof-of-concept study, adult male zebrafish (*Danio rerio*) were exposed to 5 or 25 µg/L 17β-estradiol (E2), 100 µg/L lindane, 5 nM 2,3,7,8-tetrachlorodibenzo-*p*-dioxin (TCDD) or 15 mg/L arsenic for 96 h, and the expression profiles of 59 genes involved in 7 pathways plus 2 well characterized biomarker genes, *vtg1* (*vitellogenin1*) and *cyp1a1* (*cytochrome P450 1A1*), were examined. Relative distance (RD) computational model was developed to screen favorable genes and generate appropriate gene sets for the differentiation of chemicals/concentrations selected. Our results demonstrated that the known biomarker genes were not always good candidates for the differentiation of pair of chemicals/concentrations, and other genes had higher potentials in some cases. Furthermore, the differentiation of 5 chemicals/concentrations examined were attainable using expression data of various gene sets, and the best combination was the set consisting of 50 genes; however, as few as two genes (e.g. *vtg1* and *hspa5* [*heat shock protein 5*]) were sufficient to differentiate the five chemical/concentration groups in the present test. These observations suggest that multi-parameter arrays should be more reliable for biomonitoring of chemical exposure than traditional biomarkers, and the RD computational model provides an effective tool for the selection of parameters and generation of parameter sets.

## Introduction

Increasing attention has been drawn to the wide occurrence of natural and man-made chemicals in the aquatic environment. Many chemicals can be bioaccumulated in the aquatic organisms and magnified in the food chains, thus threatening human health. The Minamata disease is a typical case, where methylmercury (MeHg) poisoning occurred in human due to the ingestion of fish and shellfish contaminated by MeHg [1]. Such scenarios have promoted researchers to develop early-warning methods for monitoring contaminants in the aquatic system through both chemical monitoring and biomonitoring.

As new pollutants in the environment are emerging rapidly, it becomes increasingly unfeasible to monitor all contaminants in the environment. Since the presence of a foreign chemical in a segment of the environment does not always indicate adverse biological effects [2], it is important to combine chemical monitoring with the biomonitoring for a reliable environmental risk assessment. An ideal approach is to examine biological responses that can reflect the contaminants in the exposed organisms [2]. Under this concept, various biomarkers from fish have been proposed and used for biomonitoring aquatic contaminants. However, most of biomarkers proposed were not specific for individual chemicals. For example, biomarker for estrogen, *vtg1* mRNA could be induced not only by the native female hormone, 17β-estradiol (E2), but also by many other compounds that can interact with estrogen receptors, including many xenobiotics, such as lindane [3]. The expression of *cyp1a1* was up-regulated by 2,3,7,8-tetrachlorodibenzo-*p*-dioxin (TCDD) as well as by other chemicals such as arsenic in mice [4].

It has been demonstrated that exposure to single chemicals generated unique gene expression signature in experimental animals [5]–[9]. Therefore, a multi-parameter quantitative real-time PCR (qRT-PCR) array could be developed as a useful tool to differentiate a complicated set of chemical groups. However, in previous studies, the parameters (genes) were selected only based on responsive difference of gene expression among chemicals after exposure [10]–[11] and did not represent the best parameter (gene) set for the discrimination of chemicals. Therefore, a proof-of-concept study was designed and conducted in the present study, with the objective of finding the best parameter (gene) set for the discrimination of chemicals tested. Especially, a relative distance (RD) computational model was developed to select gene sets from 61 gene examined for chemical discrimination. Therefore, it is feasible to integrate qRT-PCR arrays and RD computational model to develop a reliable biomonitoring tool for chemical exposure.

## Materials and Methods

### Chemicals and reagents

E2, lindane, TCDD and arsenic (Na_2_HAsO_4_·7H_2_O) were purchased from Sigma (St. Louis, MO, USA). Arsenic was dissolved in deionized water directly and the other three chemicals were dissolved in dimethyl sulfoxide (DMSO) as stock solutions. The TRizol reagent and LightCycle FastStart DNA Master SYBR Green I were obtained from Invitrogen (New Jersey, NJ, USA) and Roche Applied Science (Mannheim, Germany), respectively.

### Fish and chemical exposure

In this study, experimental procedures were carried out following the approved protocol by Institutional Animal Care and Use Committee of National University of Singapore (Protocol 079/07). Adult male zebrafish (*Danio rerio*, 5-month old) were purchased from a local aquarium farm (Mainland Tropical Fish Farm, Singapore), and acclimated for at least two weeks in our aquarium before chemical treatment. After acclimation, fish were exposed to 5 nM TCDD, 5 µg/L E2, 50 µg/L E2, 100 µg/L lindane or 15 mg/L arsenic for 96 h in a static condition. Each tank (5 L size) included 3 L exposure solution and 3 fish, and each concentration included 3 replicated tanks. During the exposure period, fish were fed once a day with commercial frozen bloodworms (Hikari) as described before [12]. The concentrations of these chemicals were chosen based on previous studies of ours and others [12]–[16], where biological effects of these concentrations have been confirmed by significant changes of some mRNAs examined. For E2, two concentrations were used to test the feasibility to develop a gene expression based model to differentiate exposure concentrations besides different chemicals. Fresh chemical solutions were daily replaced during the exposure experiment. For E2, lindane and TCDD exposure experiments, treatment and control groups received 0.01% DMSO, and for arsenic exposure experiments, treatment and control groups received 0.01% deionized water in this study. After 96-h exposure, the fish were anesthetized with MS-222 (1 mM) and livers were collected and preserved in TRizol reagent at –80°C until RNA isolation.

### Selection of target genes for PCR array

A PCR array of sixty-one zebrafish genes was designed as follows. First, seven well characterized pathways commonly affected by chemicals were selected: oxidative and metabolic stress [17]–[18], apoptosis signaling [19]–[20], proliferation and carcinogenesis [21]–[22], DNA damage and repair [23]–[24], growth arrest and senescence [25]–[26], heat shock [27]–[28], and inflammation pathways [29]–[30]. Representative genes from these pathways were selected by referring Molecular Toxicology PathwayFinder PCR array from SABioscience Gene Network Central (http://www.sabiosciences.com/rt_pcr_product/HTML/PAHS-3401Z.html). Second, annotated zebrafish orthologues of human genes were searched from Ensemble website and confirmed using online synteny tool [31]; unannotated zebrafish orhologues were manually determined first by amino acid sequence comparison with human candidate sequences through UCSC website (http://genome.ucsc.edu/) and then confirmed by comparison of genomic organization, chromosomal locations and chromosomal synteny analysis as conducted in a previously study [32]. Finally the zebrafish orthologues of 59 human genes were obtained for designing of PCR primers. In addition, two well-established biomarker genes, *vtg1* and *cyp1a1*, were also included in order to compare the potentials of biomonitoring between traditional biomarkers and genes/gene sets developed in this study, as inducers of *vtg1* and *cyp1a1* such as E2 and TCDD were also used in the present exposure experiments. The complete list of genes in PCR array and their PCR primeer sequences are presented in Table S1. The number of genes in each pathway was 14, 10, 10, 6, 4, 13 and 2 for oxidative and metabolic stress, apoptosis signaling, DNA damage and repair, proliferation and carcinogenesis, growth arrest and senescence, heat shock and inflammation pathways, respectively.

### Quantitative real-time PCR (qRT-PCR)

Total RNA was isolated from zebrafish livers with TRizol reagent and used for cDNA synthesis. Real time qPCR was performed using the LightCycler system (Roche Applied Science, Mannheim, Germany) with LightCycler FastStart DNA Master SYBR Green I following manufacturer’s instruction. The primer sequences were designed using Primer 3 software (http://frodo.wi.mit.edu/as). The amplicon efficiencies of primers were >90%. Three housekeeping genes, *β-actin* (*beta-actin*), *β-2m* (*beta-2-microglobulin*) and *rpl13a* (r*ibosomal protein L13a*), were used as internal control and the geometric means the expression of the three housekeeping genes were used as the normalized factor by 2^−ΔΔCt^ method. Each group included three biological replicates and each replicate included a pool of three fish.

### Statistical analysis

Gene expression values were logarithmically transformed (log2) before statistical analysis. The homogeneity and normality of data were examined using the Kolmogorov-Smirnov and Levene’s test, respectively. Statically significant differences between treatment and corresponding control groups were evaluated by ANOVA based on a *p*-value <0.05. Average linkage (*p* < 0.05) was used to examine the cluster relationships of different treatment groups based on mRNA expression profiles. The statistical analyses were performed using Kyplot Demo 3.0 software (Tokyo, Japan).

### Relative distance (RD) computational model

The differentiation of two chemical/concentration groups not only depends on Euclidean distance between the two groups but also depends on the distance among individual replicates within each group. In this study, the RD computational model was developed to quantitatively describe the potential that three biological replicates from group A can be differentiated from the three replicates in group B based on mRNA expression profiles (fold change), and RD between one replicate from group A treatment and three replicates from group B (*rd_a1b_*)
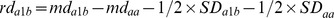
(1)

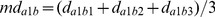
(2)

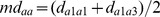
(3)

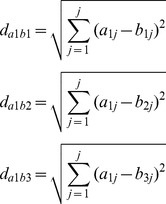
(4)

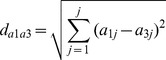
(5)

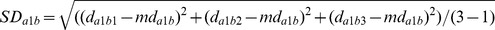
(6)

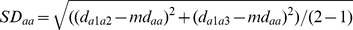
(7)


where *j* is the total number of genes examined; *a* and *b* are gene expression values in the treatment groups A and B, respectively; *md_a1b_* is the mean Euclidean distance between one biological replicate from treatment group A (*a_1_*) and three replicates from treatment group B (*b_1_, b_2_, b_3_*); *md_aa_* is the mean Euclidean distance between one biological replicate from treatment group A (*a_1_*) and other two biological replicates from the same group (*a_2_*, *a_3_*); *SD_a1b_* is the standard deviation of Euclidean distance between one biological replicate from treatment group A (*a_1_*) and three replicates from treatment group B (*b_1_, b_2_, b_3_*); *SD_aa_* is the standard deviation of Euclidean distance between one biological replicate from treatment group A treatment and other two biological replicates from the same group; *d_a1b1_*, *d_a1b2_* and *d_a1b3_* are the Euclidean distance between one biological replicate from treatment group A (*a_1_*) and three replicates from treatment group B (*b_1_, b_2_, b_3_*); *d_a1a2_* and *d_a1a3_* are the Euclidean distance of biological responses between one biological replicate from treatment group A (*a_1_*) and other two biological replicates from the same group (*a_2_*, *a_3_*).

In this study, first, we calculated all the RD values between two chemical treatment groups using expression data of individual genes. When all six RD values were >0 for each pair of chemicals, it was considered that the gene could be used to differentiate the two chemicals/concentrations. The cluster analyses (average linkage) were performed using commercial software (Kyplot Demo 3.0, Tokyo, Japan) (*p*-value <0.05) to confirm the feasibility of RD model in screening genes for the differentiation of chemical/concentration treatments. Second, the mean RD values were calculated to quantitatively compare the potentials of individual genes in differentiating two chemicals/concentrations. Finally, a C-language computational program (see Program S1) was edited for selecting genes and generating gene sets that could be used to differentiate all of five chemical/concentration treatments simultaneously using the RD model developed in this study, and maximum mean RD of each gene sets with the same amount of genes and the corresponding components of genes were outputted.

## Results

### Broad changes of gene expression patterns in the seven selected pathways in response to chemical insults

**Figure 1:**
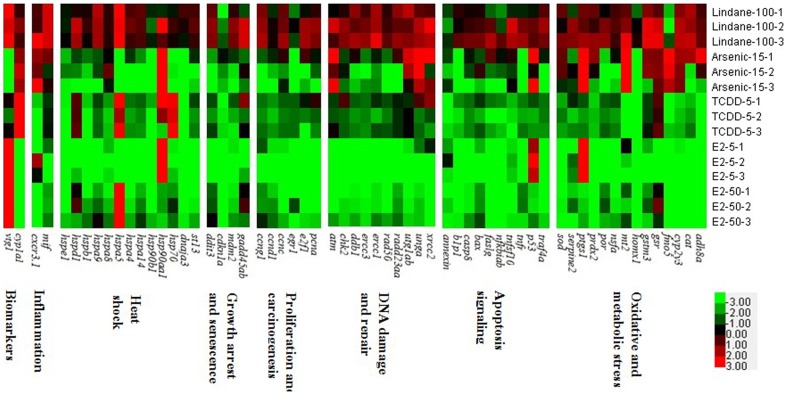
Gene expression profiles included in seven selected pathways in male zebrafish livers after exposure to 100 µg/L lindane, 5 nM 2,3,7,8-tetrachlorodibenzo-*p*-dioxin (TCDD), 5 µg/L 17β-estradiol (E2), 25 µg/L E2, or 15 mg/L arsenic for 96 h. There were 3 biological replicates, and each replicate were pooled from 3 fish. Gene expressions were expressed as fold change relative to the corresponding control. The full names of genes can be found in Tables S1 or S2.

Adult male zebrafish were treated with 5 nM TCDD, 5 µg/L E2, 50 µg/L E2, 100 µg/L lindane or 15 mg/L arsenic for 96 hours and no mortalities were observed throughout the exposure experiment. As shown in Figure 1 and Table S2, exposure to different chemicals led to different gene expression profiles. TCDD exposure significantly down-regulated the expression of most selected genes involved in the oxidative and metabolic stress, apoptosis signaling, DNA damage and repair, proliferation and carcinogenesis, growth arrest and senescence, heat shock and inflammation pathways, while the expression of *cyp1a1*, *hspa5* and *hsp70* (*heat shock protein 70-kDa*) was among the highest up-regulated. Treatment with arsenic significantly altered the expression of most selected genes in the seven pathways, such as up-regulation of expression of *ptgs1* (*prostaglandin-endoperoxide synthase 1*), *cyp1a1* and *hsp90aa1* (*heat shock protein 90, alpha, class A member 1, tandem duplicate 1*), and down-regulation of *b1p1* (*Bcl-XL-like protein 1*), *tnfr* (*tumor necrosis factor receptor*) and *vtg1*. A significant up-regulation in the expression of *vtg1* was observed upon exposure to 5 or 50 µg/L E2, clearly showing estrogenic activity. Similar to TCDD, exposure to E2 (5 or 50 µg/L) significantly down-regulated the expression of most selected genes included in the seven pathways investigated. In contrast, exposure to lindane up-regulated the expression of most selected genes in the seven pathways; with exception of only few down-regulated genes, notably *cdkn1a* (*cyclin-dependent kinase inhibitor 1A, transcript variant 1*) in the growth arrest and senescence pathway and *fmo5* (*flavin containing monooxygenase 5*) in the oxidative and metabolic stress pathway.

### Correlation of RD and potential differentiation of chemical treatment pairs

**Figure 2:**
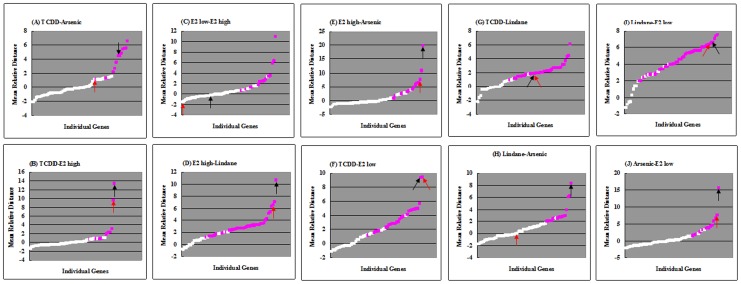
Mean Relative Distances (RDs) between two chemicals/concentration groups. (A) TCDD vs. Arsenic; (B) TCDD vs. E2_high; (C) E2_low vs. E2_high; (D) E2_high vs. Lindane; (E) E2_high vs. Arsenic; (F) TCDD vs. E2_low; (G) TCDD vs. Lindane; (H) Lindane vs. Arsenic; (I) Lindane vs. E2_low; (J) Arsenic vs. E2_low. Black arrows indicate the positions of *vtg1*, and red arrows indicate the positions of *cyp1a1*; White boxes indicate the positions of genes that did not pass the model test and could not be used to discriminate the corresponding two chemicals/concentrations; Pink boxes indicate the positions of genes that passed the model test and could be used to discriminate the corresponding two chemicals/concentrations. TCDD: 5 nM 2,3,7,8-tetrachlorodibenzo-*p*-dioxin; lindane: 100 µg/L lindane; arsenic: 15 mg/L arsenic; E2_low: 5 µg/L 17β estradiol; E2_high: 50 µg/L 17β-estradiol. The information of RDs and the corresponding genes can be found in Table S3.

Using an RD computational model, we calculated all of RD values between two chemical/concentration treatment groups based on expression fold change of individual genes and the results are presented in Figure 2 (see details in Table S3) for all of the 10 possible chemical/concentration pairs. The ability of each of the 61 genes to discriminate the chemical/concentration pairs was tested by the software Kyplot Demo 3.0 program and the findings are presented in Figure 2. There was a good correlation of the RD and the ability to discriminate pair of chemicals/concentrations. All the genes with top and high RD values were found to be able to discriminate pair of chemicals/concentrations. For example, the two best known biomarker genes, *vtg1* and *cyp1a1*, were able to discriminate eight of the ten pairs: TCDD/arsenic, TCDD/E2_high, E2_high/lindane, E2_high/arsenic, TCDD/E2_low, TCDD/lindane, lindane/E2_low, and arsenic/E2_low. However, for the lindane/arsenic pair, *cyp1a1* could not be used to discriminate them, while for the E2_low/E2_high concentration pair, both *vtg1* and *cyp1a1* failed to discriminate them. Interestingly, *vtg1* and *cyp1a1* were not always among the top of the list based on the calculated RD. There were also many other genes (even with better RD) that could be also used to differentiate the corresponding pair of chemicals.

### Selection of discriminating gene sets based on RD computational model

**Figure 3:**
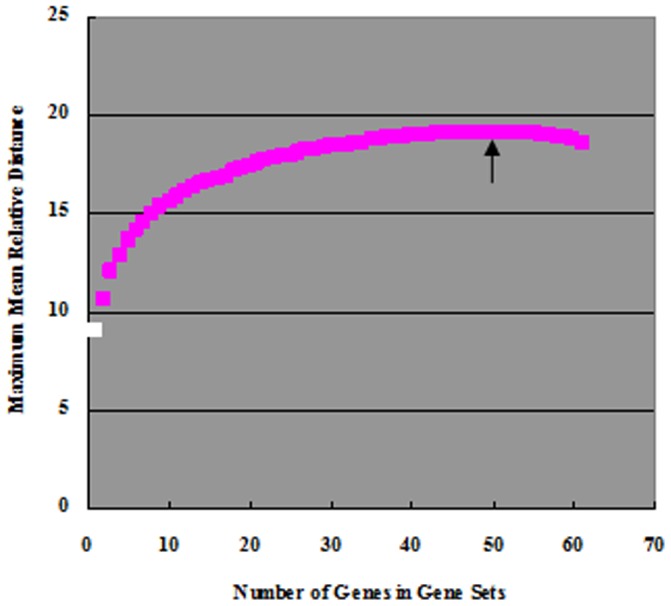
Maximum mean RD of gene sets with different numbers of genes among 5 chemicals/concentrations. Black arrow indicates the position of gene set (50 genes), where maximum RD was achieved. White box indicates the position of gene set (1 gene) that did not pass the model test and could not be used to differentiate the corresponding five chemicals/concentrations; Pink boxes indicate the positions of gene sets that passed the model test and could be used to differentiate the corresponding five chemicals/concentrations. The information about maximum mean RDs and the corresponding components of genes can be found in Table S4.

**Figure 4:**
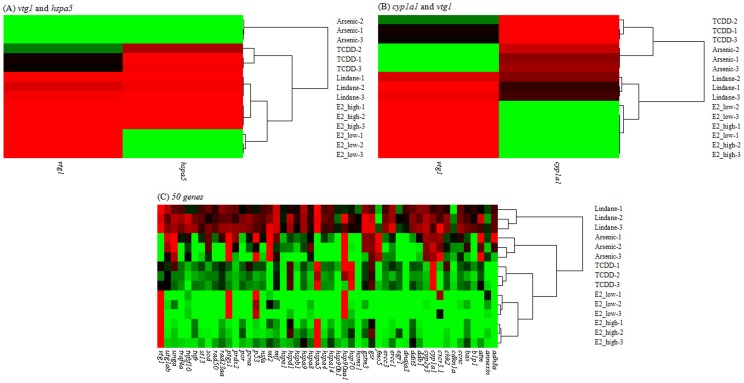
Clustering relationships among chemicals/concentrations using mRNA expression data of (A) *cyp1a1* and *vtg1*, (B) *vtg1* and *hspa5* and (C) 50 genes with the marximum RD. TCDD: 5 nM 2,3,7,8-tetrachlorodibenzo-*p*-dioxin; lindane: 100 µg/L lindane; arsenic: 15 mg/L arsenic; E2_low: 5 µg/L 17β-estradiol; E2_high: 50 µg/L 17β-estradiol. The full names of genes can be found in Table S1 or S2.

While it is relatively easy to discriminate a pair of chemical treatment groups based on expression data from one or few genes, it is more challenging to discriminate multiple treatment groups. In the current dataset, no single gene can be used to discriminate all of the five chemical/concentration groups. Thus, it was necessary to select a gene set for discriminating the chemical/concentration groups. Here, we further explored the RD model to select best gene sets for differentiating all of the five chemical/concentration groups. RDs were computed for all possible gene combinations from one to 61 genes and the highest mean distances for gene sets from 1 to 65 genes are presented in Figure 3. For example, the 2-gene set of the highest mean RD was *vtg1* and *hspa5* with a value of 10.57 (Figure 3 and Table S4) and the two genes can be used to discriminate the five chemical/concentration groups perfectly (Figure 4A). In comparison, the gene pair of best known biomarkers, *vtg1* and *cyp1a1*, has a value of 10.33 and they could not correctly discriminate all of the five groups, particularly the two concentration groups of E2 treatment (Figure 4B). All other gene sets (3 or more genes) of the highest mean RD were also capable of differentiating all the five chemical/concentration groups correctly (Figure 3). In general, there was an increase of mean RD with the number of genes in gene sets and the maximal mean RD (19.153) was observed in the set with 50 genes, where chemicals were also completely differentiated, including different concentrations (Figure 4C).

## Discussion

The environment is continuously loaded with natural and man-made chemicals, and the effects of contaminant exposure to human health have been extensively documented [33]–[37]. In general, adverse effects of contaminants at population levels in wildlife and human tend to be delayed; when the effects finally become clear, the destructive processes may have been beyond the point where it can be reversed by available remedial actions [2]. Therefore, various biomonitoring methods have been developed in the past few decades for the purpose of early warning. However, most of these methods focused on one or several biological parameters (e.g., biomarkers vitellogenins and cytochrome P450 enzymes 1A1) [38]–[43]. To search for more biomarker genes to predict chemical contamination, it is common to use high throughput and large scale analyses such as DNA microarray and more recently RNA-seq platform [8], [12], [44]. However, the methodology for selecting biomarkers from thousands of genes could be a great challenge. Here we performed a proof-of-concept study by selecting a handful of biomarker genes to develop a practical assay with the aid of RD computational model.

Here four chemicals including E2, lindane, TCDD and arsenic were tested. Both E2 and lindane exposures caused up-regulation of hepatic *vtg1* expression; similarly, treatment with TCDD or arsenic showed up-regulation of *cyp1a1* expression. These observations are consistent with previous studies [3]–[4], [45], suggesting the effectiveness of these chemical exposure experiments. In general, exposure to different chemicals resulted in different gene expression profiles in the seven biological pathways examined. For example, both of E2 and lindane induced *vtg1* expression, but E2 down-regulated the expression of essentially all of the selected genes in the seven pathways while lindane up-regulated the expression of most of these genes. Similarly, TCDD down-regulated the expression of most of genes and arsenic up-regulated many of the genes, especially in two pathways, oxidative_and_metabolic_stress and DNA_damage_and_repair, suggesting a molecular basis for their discrimination.

In the current data set, we found that none of the 61 genes could be used to correctly discriminate all of the five chemical/concentration groups; thus, it has to rely on multiple gene sets for successful discrimination, which should be the direction for future development of multiple gene signatures for discrimination of a multiple chemical groups, as previously proposed [8], [12]. To systematically select the best discriminator genes, here we developed a computational model using RD to determine the prediction power of each gene or in combination with others. First, we demonstrated that there was a positive correlation between the RD values and the discrimination of different treatments groups (Fig. 2). In our data set, a minimum of two genes (e.g. *vtg1* and *hspa5*) could be used to successfully discriminate all of the five chemical/concentration groups. There is a general increase of mean RD values with the number of genes added to the gene set, which indicate the power of using more genes for discriminating more complicated data set. In our dataset, we also found that the 50-gene set had the highest mean RD values, indicating that there is an optimal gene number used for the discrimination. From a practical viewpoint, the used of minimal number of genes will minimize workload and ease downstream data analysis. However, using more genes, especially those representing different molecular pathways, provides additional important biological information in molecular-marker based biomonitoring.

In summary, the data of this study demonstrated chemicals that induced similar responses in biomarker (e.g., TCDD and arsenic, E2 and lindane) could cause different biological responses depending on the parameters examined, and the use of parameter sets consisting of different biological responses for biomonitoring should be more appropriate. Furthermore, the computational model based on RD may be useful to select appropriate gene sets to develop efficient biomarker-based biomonitoring. Considering the rapid, sensitive, convenient and high-throughput properties of PCR, a PCR array including multiple gene parameters should be a feasible tool to develop for biomonitoring of chemical exposure.

## Supporting Information

Table S1 **Sequences of primers for selected genes.**
(DOC)

Table S2 **mRNA expression profiles in the livers of zebrafish after chemical exposure.**
(DOC)

Table S3 **Mean relative distances (MRDs) of individual genes between chemicals.**
(DOC)

Table S4 **Maximum mean relative distances (MMRDs) of gene sets with different amounts of genes among 5 chemicals/concentrations and the corresponding components of genes.**
(DOC)

Program S1 (ZIP)
