# Supplementary material for: Selection of Reliable Biomarkers from PCR Array Analyses Using Relative Distance Computational Model: Methodology and Proof-of-Concept Study
Source: PLoS One. 2013 Dec 12;8(12):e83954. doi: 10.1371/journal.pone.0083954 (PMC3861511; doi:10.1371/journal.pone.0083954)
Supplement: Table S1 — Sequences of primers for selected genes. (DOC) [file pone.0083954.s001.doc]

**Table S1 Sequences of primers for selected genes**.

| Pathway | Gene name (Abbreviation) | Sequence of the primers (5’-3’) | Accession number |
| --- | --- | --- | --- |
| Oxidative  and  metabolic  stress | *alcohol dehydrogenase 8a* (*adh8a*) | Forward: ggactttgtcacaccgacct  Reverse: gagaggaagagggggatgac | NM_001001946 |
|  | *catalase* (*cat*) | Forward: agatgaaactgtggaaggagggtc  Reverse: aaacactttggctttggagtagcg | AF170069 |
|  | *cytochrome p450 cyp2y3* (*cyp2y3*) | Forward: gaccaccagcacaactctga  Reverse: ccatatagcgctgcacttca | AY927850 |
|  | *flavin containing monooxygenase 5* (*fmo5*) | Forward: aatgtcagcatcatgaaaatgtcagaattg  Reverse: tgcttcacagacatatattacttaacagac | BC153910 |
|  | *glutathione reductase* (*gsr*) | Forward: acagtcagtgaggatgatgtgccag  Reverse: tagacccaagagtggaaagaataccagc | NM_001020554 |
|  | *glutathione S-transferase M3* (*gstm3*) | Forward: gctggggacaagatcacatt  Reverse: tttggccatcttgttgttca | NM_001162851 |
|  | *heme oxygenase 1* (*homx1*) | Forward: aaagcaaagcggcagagaac  Reverse: tcctgagggaagtaaatgggc | NM_001127516 |
|  | *metallothionein 2* (*mt2*) | Forward: tgtgccaagactggaacttg  Reverse: cttcattgacagcagctgga | NM_001131053 |
|  | *N-ethylmaleimide-sensitive factor a* (*nsfa*) | Forward: ctgtccattgggcaagaaat  Reverse: tgaactcagcagccattttg | NM_001044328 |
|  | *NADPH-cytochrome P450 oxidoreductase (por)* | Forward: accctacagacaacgctcag  Reverse: aatactcacacaccgcaggc | AY949986 |
|  | *peroxiredoxin 2* (*prdx2*) | Forward: ttcacctttgtgtgtcccac  Reverse: tgcctcaagatgcccttatc | NM_001002468 |
|  | *prostaglandin-endoperoxide synthase 1* (*ptgs1*) | Forward: atgtgactgcaccagaacgg  Reverse: actttccgcatgagccaatc | NM_153656 |
|  | *serine proteinase inhibitor, clade E, member 2* (*serpine2*) | Forward: ccaacgcactgttccctaat  Reverse: catatccgcagtcaccacac | NM_200184 |
|  | *Cu/Zn-superoxide dismutase* (*sod*) | Forward: aagaagccagtgaaggtgact  Reverse: acattacccaggtctccgac | Y12236 |
| Apoptosis  signaling | *tnf receptor-associated factor 4a* (*traf4a*) | Forward: actgaagcagcagcaatgcc  Reverse: aatccaatgggagctggtcc | NM_205762 |
|  | *tumor suppressor p53* (*p53*) | Forward: atcatctgagcccaaacagg  Reverse: aaatgacccctgtgacaagc | U60804 |
|  | *tumor necrosis factor (ligand) superfamily, member 10* (*tnfsf10*) | Forward: agatccagtcttgggagtccg  Reverse: aacatactgcaacatcggtttcc | NM_001002593 |
|  | *TNF receptor* (*tnfr*) | Forward: accatcagcctcaaatagcac  Reverse: tcctcctctttctccagtcctg | AF250042 |
|  | *nuclear factor of kappa light polypeptide gene enhancer in B-cells inhibitor, alpha b* (*nfkbiab*) | Forward: tgcaggaaaggatctctggc  Reverse: ttcgatgagaagtttgaccatgtc | NM_199629 |
|  | *fas ligand* (*faslg*) | Forward: agctgacacaggaattgccc  Reverse: atgcgaaagaaactccacgc | NM_001042701 |
|  | *bax* (*bax*) | Forward: ggagatgagctggatggaaa  Reverse: aggcgacaggcaaagtagaa | AF231015 |
|  | *caspase-8* (*casp8*) | Forward: ctcaaacgaacaggcactga  Reverse: acaaaagcacccattgaagc | AF273220 |
|  | *Bcl-XL-like protein 1* (*b1p1*) | Forward: agagcgtgatggatgaggtg  Reverse: tcttgcgatttcctgctttcc | AF317837 |
|  | *annexin* (*annexin*) | Forward: aaagtgatgctcaggctctg  Reverse: tatttgttggaggcttcgtg | Y16043 |
| Proliferation  and  carcinogenesis | *proliferating cell nuclear antigen* (*pcna*) | Forward: ggcaacatcaagctctcaca  Reverse: tgcaattttgtcctcaacca | AF140608 |
|  | *E2F transcription factor 1* (*e2f1*) | Forward: atcatctccactcctggcac  Reverse: ttcagagacgtgtcgtagcg | ENSDART00000109944 |
|  | *early growth response 1* (*egr1*) | Forward: aaacccatccagacacaagc  Reverse: ttggccggttaggatacttg | NM_131248 |
|  | *cyclin C* (*ccnc*) | Forward: atgtggcgtgtgtggttcag  Reverse: aggaggtttgggtttgggca | NM_199951 |
|  | *cyclin D1* (*ccnd1*) | Forward: tgacttgccttgacttgtcg  Reverse: gaaaaagcagggagcacttg | NM_131025 |
|  | *cyclin G1* (*ccng1*) | Forward: aactggaaggtcaaggctcc  Reverse: agaagagacaaagcaagcagg | AY423016 |
| House  keeping  genes | *beta-actin* (*β-actin*) | Forward: ctcttccagccttccttcct  Reverse cttctgcatacggtcagcaa | AF057040 |
|  | *ribosomal protein L13a* (*rpl13a*) | Forward: tcccagctgctctcaagatt  Reverse: ttcttggaatagcgcagctt | NM_212784 |
|  | *beta-2-microglobulin* (*β-2m*) | Forward: aaccaaacaccctgatctgc  Reverse caacgctctttgtgaggtga | L05383 |

**Table S1 (*Continued***)

| Pathway | Gene name (Abbreviation) | Sequence of the primers (5’-3’) | Accession number |
| --- | --- | --- | --- |
| DNA  damage  and  repair | *X-ray repair complementing defective repair in Chinese hamster cells 2* (*xrcc2*) | Forward: acagcacgagtcaggatggc  Reverse: agacctccactgtgcgttgg | ENSDART00000005804 |
|  | *UDP glucuronosyltransferase 1 family a, b* (*utg1ab*) | Forward: tgctcagcgtttggtgtctc  Reverse: atccagttcaggtcatgggc | NM_213422 |
|  | *uracil-DNA glycosylase a* (*unga*) | Forward: aaatcaaggtgtcctgctgc  Reverse: tacaaccgaagaatccacgg | NM_200974 |
|  | *RAD50 homolog* (*rad50*) | Forward: agacatgggcaacatccgac  Reverse: acttctcgtcgctcattgcg | ENSDART00000075927 |
|  | *RAD23 homolog Aa* (*rad23aa*) | Forward: agacattgcagcagcagacc  Reverse: tctggatggttctgagggtg | NM_001003739 |
|  | *excision repair cross-complementing rodent repair deficiency, complementation group 1* (*ercc1*) | Forward: ttggagggcatcattaaagc  Reverse: gcctgatggtctcagatggt | NM_001103138 |
|  | *excision repair cross-complementing rodent repair deficiency, complementation group 3* (*ercc3*) | Forward: aggaggaggaggaagagacg  Reverse: ggcttcaggtccatgttgat | NM_201582 |
|  | *damage specific DNA binding protein 1* (*ddb1*) | Forward: gtggaccgcagagaagtagc  Reverse: tctccactgcactcatccag | NM_200626 |
|  | *protein kinase Chk2* (*chk2*) | Forward: tcatctgtttgggtgggtatcc  Reverse: tttccttgttgcttctggacg | AF265346 |
|  | *ataxia telangiectasia mutated* (*atm*) | Forward: ggatttgttctgtgggctgt  Reverse: tgctgttgctcagagcctta | FJ345409 |
| Growth  Arrest  And  senescence | *growth arrest and DNA-damage-inducible, alpha, b* (*gadd45ab*) | Forward: aaaggcactggaagaggtcc  Reverse: acagaaggcttgaatcagggt | NM_001002216 |
|  | *mdm2 homolog* (*mdm2*) | Forward: agtgaagagagcgaagactcag  Reverse: aagaggagggttgaactggtc | AF010255 |
|  | *cyclin-dependent kinase inhibitor 1A, transcript variant 1* (*cdkn1a*) | Forward: cgcaaacagaccaacatcac  Reverse: aacgctgctacgagacgaat | XM_001923789 |
|  | *DNA-damage-inducible transcript 3* (*ddit3*) | Forward: atatactgggctccgacacg  Reverse: ttcgttcttcttgccttggt | NM_001082825 |
| Heat  shock | *suppression of tumorigenicity 13* (*st13*) | Forward: ttctgaccaaactctggacg  Reverse: atggatgctggaactctgtg | NM_199769 |
|  | *DnaJ (Hsp40) homolog, subfamily A, member 3A* (*dnaja3*) | Forward: tttgcacagctagcggaagc  Reverse: attgaagatggcgttgaaatcc | NM_201313 |
|  | *heat shock cognate 70 kDa protein* (*hsp70*) | Forward: cctatggcctggacaagaaa  Reverse: atgggtgaccatgcgattat | NM_001200012 |
|  | *heat shock protein 90, alpha (cytosolic), class A member 1, tandem duplicate 1* (*hsp90aa1*) | Forward: tgtcaagccagacttcggtg  Reverse: tcgccttcctcaagatccac | NM_131328 |
|  | *heat shock protein 90, beta (grp94), member 1* (*hsp90b1*) | Forward: tgttgacattgatggcacag  Reverse: ttcattggtcagagacagcag | NM_198210 |
|  | *heat shock protein 4* (*hspa4*) | Forward: cctggaactgaggaaatgga  Reverse: ctccacgaacagattcagca | BC065970 |
|  | *heat shock protein 14* (*hspa14*) | Forward: tgtgtttaaggatggacgagc  Reverse: tgcatctgggtcatcatagc | NM_001045076 |
|  | *heat shock protein 5* (*hspa5*) | Forward: acgagaacacagaagacggg  Reverse: atcccaatcactgtcccaac | NM_213058 |
|  | *heat shock protein 8* (*hspa8*) | Forward: aggtcgactaagccattcca  Reverse: tctgttccctgtgatctgga | ENSDART00000099994 |
|  | *heat shock protein 9* (*hspa9*) | Forward: tgatggagagcggcttgtag  Reverse: tttgccatggacttctagcc | NM_201326 |
|  | *heat shock protein, alpha-crystallin-related, 1* (*hspb1*) | Forward: acactggccagggtatatgc  Reverse: aagcatctggaaatgaagcc | NM_001008615 |
|  | *heat shock 60kD protein 1* (*hspd1*) | Forward: tgagacagatgaggcctgtg  Reverse: ccagctctgctcaatgatga | NM_181330 |
|  | *heat shock 10 protein 1* (*hspe1*) | Forward: agaccgtgtcaagaggaggc  Reverse: acacagatggctggacaagc | NM_131526 |
| Inflammation | *macrophage migration inhibitory factor* (*mif*) | Forward: tttgttcatctctgtcgcagtc  Reverse: acgccgaggtgtttgttgag | NM_001043321 |
|  | *chemokine (C-X-C motif) receptor 3.1* (*cxcr3.1*) | Forward: agaagaagagggccatccat  Reverse: caacatccaacgtggttctg | NM_001089430 |
| Biomarkers | *vitellogenin 1* (*vtg1*) | Forward: ctgcgtgaagttgtcatgct  Reverse: gaccagcattgcccataact | NM_001044897 |
|  | *cytochrome P450 1A1* (*cyp1a1*) | Forward: cctgggcggttgtctatcta  Reverse: tgaggaatggtgaagggaag | AF210727 |
