# Supplementary material for: Selection of Reliable Biomarkers from PCR Array Analyses Using Relative Distance Computational Model: Methodology and Proof-of-Concept Study
Source: PLoS One. 2013 Dec 12;8(12):e83954. doi: 10.1371/journal.pone.0083954 (PMC3861511; doi:10.1371/journal.pone.0083954)
Supplement: Table S2 — mRNA expression profiles in the livers of zebrafish after chemical exposure. (DOC) [file pone.0083954.s002.doc]

**Table S2 mRNA expression profiles in the livers of zebrafish after chemical exposure^a,b,c^**.

| Pathways | Gene Name (Abbreviation) | Chemicals | | | | |
| --- | --- | --- | --- | --- | --- | --- |
|  |  | TCDD | Lindane | Arsenic | E2_low | E2_high |
| Oxidative  and  metabolic  stress | *alcohol dehydrogenase 8a* (*adh8a*) | -3.74 | 1.03 | 0.44 | -7.83 | -7.20 |
|  | *catalase* (*cat*) | -1.83 | 2.02 | -2.58 | -8.52 | -5.224 |
|  | *cytochrome P450 CYP2Y3* (*cyp2y3*) | -2.36 | 1.98 | 1.65 | -3.50 | -4.05 |
|  | *flavin containing monooxygenase 5* (*fmo5*) | -4.21 | -2.69 | 2.77 | -4.25 | -5.21 |
|  | *glutathione reductase* (*gsr*) | 0.92 | 2.10 | 1.39 | -4.51 | 0.32 |
|  | *glutathione S-transferase M3* (*gstm3*) | -0.56 | 2.38 | 0.32 | -3.42 | -1.76 |
|  | *heme oxygenase 1* (*homx1*) | -7.90 | -0.30 | -4.26 | -8.42 | -5.43 |
|  | *metallothionein 2* (*mt2*) | -3.77 | 1.82 | 3.81 | -1.95 | -1.44 |
|  | *N-ethylmaleimide-sensitive factor a* (*nsfa*) | -1.41 | 0.84 | -2.30 | -5.59 | -2.66 |
|  | *NADPH-cytochrome P450 oxidoreductase* (*por*) | -1.26 | 1.09 | -4.28 | -8.16 | -1.55 |
|  | *peroxiredoxin 2* (*prdx2*) | -2.37 | 1.16 | -0.07 | -5.76 | -5.08 |
|  | *prostaglandin-endoperoxide synthase 1* (*ptgs1*) | -1.46 | 1.42 | 5.54 | 4.99 | -2.41 |
|  | *serine proteinase inhibitor, clade E, member 2* (*serpine2*) | -1.51 | 0.85 | -0.87 | -1.90 | -2.18 |
|  | *Cu/Zn-superoxide dismutase* (*sod*) | -1.82 | 0.95 | 0.6 | -4.88 | -3.37 |
| Apoptosis  signaling | *tnf receptor-associated factor 4a* (*traf4a*) | -1.56 | 1.44 | -3.15 | -7.08 | -3.19 |
|  | *tumor suppressor p53* (*p53*) | -1.57 | 0.64 | 3.02 | 3.45 | -2.22 |
|  | *tumor necrosis factor (ligand) superfamily, member 10* (*tnfsf10*) | -2.81 | 1.38 | -0.14 | -2.40 | -4.27 |
|  | *TNF receptor* (*tnfr*) | -1.66 | 0.93 | -4.42 | -7.70 | -1.85 |
|  | *nuclear factor of kappa light polypeptide gene enhancer in B-cells inhibitor, alpha b* (*nfkbiab*) | -2.28 | -0.5 | -1.69 | -8.73 | -4.18 |
|  | *fas ligand* (*faslg*) | -2.28 | 0.66 | -1.14 | -4.08 | -3.84 |
|  | *bax* (*bax*) | -1.26 | 0.82 | -0.86 | -3.09 | -1.43 |
|  | *caspase-8* (*casp8*) | -1.50 | 0.79 | -1.80 | -4.57 | -2.42 |
|  | *Bcl-XL-like protein 1* (*b1p1*) | -2.20 | 0.60 | -5.92 | -6.52 | -3.34 |
|  | *annexin* (*annexin*) | -3.77 | -1.08 | -1.28 | -1.60 | -2.78 |
| DNA  damage  and  repair | *X-ray repair complementing defective repair in Chinese hamster cells 2* (*xrcc2*) | -0.34 | 1.94 | 1.69 | -3.35 | -3.50 |
|  | *UDP glucuronosyltransferase 1 family a, b* (*utg1ab*) | -0.17 | 1.62 | 1.65 | -5.06 | -3.06 |
|  | *uracil-DNA glycosylase a* (*unga*) | -0.61 | 1.70 | 3.19 | -3.58 | -2.42 |
|  | *RAD50 homolog* (*rad50*) | -1.12 | 0.82 | -4.36 | -7.14 | -3.53 |
|  | *RAD23 homolog Aa* (*rad23aa*) | -0.50 | 1.51 | -2.79 | -4.79 | -1.69 |
|  | *excision repair cross-complementing rodent repair deficiency, complementation group 1* (*ercc1*) | -1.23 | 2.21 | -1.20 | -4.58 | -2.32 |
|  | *excision repair cross-complementing rodent repair deficiency, complementation group 3* (*ercc3*) | -1.49 | 1.28 | -2.97 | -4.14 | -2.93 |
|  | *damage specific DNA binding protein 1* (*ddb1*) | -1.42 | 1.57 | -1.62 | -4.11 | -2.50 |
|  | *protein kinase Chk2* (*chk2*) | -0.45 | 1.24 | -0.81 | -4.88 | -3.39 |
|  | *ataxia telangiectasia mutated* (*atm*) | -0.52 | 1.13 | 3.16 | -6.47 | -2.94 |

**Table S2 (*Continued***)

| Pathways | Gene Name | Chemicals | | | | |
| --- | --- | --- | --- | --- | --- | --- |
|  |  | TCDD | Lindane | Arsenic | E2_low | E2_high |
| Proliferation  and  carcinogenesis | *proliferating cell nuclear antigen* (*pcna*) | -0.73 | 1.27 | -1.72 | -6.05 | -3.21 |
|  | *E2F transcription factor 1* (*e2f1*) | -1.97 | 1.21 | -1.68 | -4.39 | -3.53 |
|  | *early growth response 1* (*egr1*) | -1.24 | -0.78 | -5.72 | -7.27 | -3.24 |
|  | *cyclin C* (*ccnc*) | -1.31 | 1.45 | -0.55 | -3.30 | -2.80 |
|  | *cyclin D1* (*ccnd1*) | -1.95 | 0.00 | -2.49 | -5.21 | -1.81 |
|  | *cyclin G1* (*ccng1*) | -1.45 | 1.77 | -3.03 | -3.40 | -1.31 |
| Growth  arrest  and  senescence | *growth arrest and DNA-damage-inducible, alpha, b* (*gadd45ab*) | 0.06 | 1.38 | -0.84 | -3.03 | -0.23 |
|  | *mdm2 homolog* (*mdm2*) | -1.92 | 1.17 | -2.85 | -4.71 | -2.37 |
|  | *cyclin-dependent kinase inhibitor 1A, transcript variant 1* (*cdkn1a*) | -2.94 | -2.07 | -1.33 | -7.68 | -5.02 |
|  | *DNA-damage-inducible transcript 3* (*ddit3*) | -1.12 | 1.47 | -3.18 | -5.59 | -0.75 |
| Heat  shock | *suppression of tumorigenicity 13* (*st13*) | -1.34 | 0.87 | -2.99 | -5.41 | -2.60 |
|  | *DnaJ (Hsp40) homolog, subfamily A, member 3A* (*dnaja3*) | -3.48 | 0.30 | -4.11 | -5.87 | -3.63 |
|  | *heat shock 70 kDa protein* (*hsp70*) | 2.93 | 0.24 | -3.42 | -2.85 | -1.18 |
|  | *heat shock protein 90, alpha (cytosolic), class A member 1, tandem duplicate 1* (*hsp90aa1*) | 0.92 | 1.15 | 10.95 | 10.9 | -2.10 |
|  | *heat shock protein 90, beta (grp94), member 1* (*hsp90b1*) | -2.98 | -0.70 | -3.76 | -9.79 | -3.75 |
|  | *heat shock protein 4* (*hspa4*) | -1.65 | 0.97 | -4.18 | -5.97 | -2.59 |
|  | *heat shock protein 14* (*hspa14*) | -1.52 | 1.00 | -2.92 | -4.32 | -1.40 |
|  | *heat shock protein 5* (*hspa5*) | 2.88 | 3.02 | -4.39 | -5.99 | 3.72 |
|  | *heat shock protein 8* (*hspa8*) | -2.21 | 0.50 | 0.31 | -2.59 | -2.15 |
|  | *heat shock protein 9* (*hspa9*) | -1.08 | 1.99 | -1.76 | -4.97 | -1.01 |
|  | *heat shock protein, alpha-crystallin-related, 1* (*hspb1*) | -3.33 | 0.37 | -2.61 | -6.66 | -2.30 |
|  | *heat shock 60kD protein 1* (*hspd1*) | 0.96 | 1.54 | -1.81 | -3.68 | -0.34 |
|  | *heat shock 10 protein 1* (*hspe1*) | -3.30 | -0.23 | -2.20 | -4.60 | -3.17 |
| Inflammation | *macrophage migration inhibitory factor* (*mif*) | -0.42 | 2.52 | 0.39 | -3.85 | -1.78 |
|  | *chemokine (C-X-C motif) receptor 3.1* (*cxcr3.1*) | -3.90 | 1.65 | 3.13 | -0.44 | -3.40 |
| Biomarkers | *vitellogenin 1* (*vtg1*) | -0.37 | 2.93 | -6.54 | 12.05 | 14.85 |
|  | *cytochrome P450 1A1* (*cyp1a1*) | 3.77 | 1.00 | 1.99 | -8.55 | -7.14 |

^a^Values represent the mean of three replicate samples, and each sample included three fish. ^b^Gene expressions were expressed as fold change relative to the corresponding control. ^c^TCDD: 5 nM 2,3,7,8-tetrachlorodibenzo-*p*-dioxin; Lindane: 100 µg/L lindane; Arsenic: 15 mg/L arsenic; E2_low: 5 µg/L 17β-estradiol; E2_high: 50 µg/L 17β-estradiol.
