# Supplementary material for: Selection of Reliable Biomarkers from PCR Array Analyses Using Relative Distance Computational Model: Methodology and Proof-of-Concept Study
Source: PLoS One. 2013 Dec 12;8(12):e83954. doi: 10.1371/journal.pone.0083954 (PMC3861511; doi:10.1371/journal.pone.0083954)
Supplement: Table S3 — Mean relative distances (MRDs) of individual genes between chemicals. (DOC) [file pone.0083954.s003.doc]

**Table S3 Mean relative distances (MRDs) of individual genes between chemicals^a,b,c^**.

| E2_high  and TCDD | | E2_high  and lindane | | E2_high  and E2_low | | E2_high  and arsenic | | E2_low  and arsenic | |
| --- | --- | --- | --- | --- | --- | --- | --- | --- | --- |
| Genes | MRDs | Genes | MRDs | Genes | MRDs | Genes | MRDs | Genes | MRDs |
| *gadd45ab* | -1.35 | *gadd45ab* | -0.80 | *cyp1a1* | -1.50 | *rad50* | -2.25 | *ccng1* | -2.15 |
| *fmos* | -1.01 | *hspd1* | -0.61 | *adh8a* | -1.29 | *egr1* | -1.83 | *hspa4* | -1.95 |
| *e2f1* | -0.85 | *hspa5* | -0.57 | *mt2* | -1.15 | *hspb1* | -1.25 | *p53* | -1.81 |
| *serpine2* | -0.83 | *egr1* | -0.30 | *e2f1* | -0.98 | *st13* | -1.18 | *mdm2* | -1.73 |
| *hspa5* | -0.81 | *fmo5* | -0.08 | *serpine2* | -0.97 | *e2f1* | -1.08 | *cxcr3.1* | -1.70 |
| *hsp90b1* | -0.76 | *gsr* | -0.07 | *chk2* | -0.88 | *traf4a* | -1.07 | *hsp90aa1* | -1.46 |
| *hspb1* | -0.69 | *hsp70* | 0.17 | *hspe1* | -0.77 | *hspa9* | -1.07 | *egr1* | -1.40 |
| *mdm2* | -0.66 | *serpine2* | 0.60 | *utg1ab* | -0.74 | *mdm2* | -1.06 | *b1p1* | -1.37 |
| *bax* | -0.58 | *annexin* | 0.62 | *xrcc2* | -0.71 | *ccnd1* | -1.06 | *rad23aa* | -1.37 |
| *tnfr* | -0.56 | *cdkn1a* | 0.70 | *ccng1* | -0.56 | *cat* | -1.05 | *rad50* | -1.24 |
| *cxcr3.1* | -0.54 | *ccnd1* | 0.72 | *prdx2* | -0.51 | *gadd45ab* | -1.04 | *ccnd1* | -1.23 |
| *hspe1* | -0.53 | *tnfr* | 0.79 | *cyp2y3* | -0.50 | *ddb1* | -1.01 | *cat* | -1.16 |
| *hspa9* | -0.52 | *hsp90b1* | 1.02 | *hspa8* | -0.48 | *nsfa* | -1.00 | *e2f1* | -1.06 |
| *ddit3* | -0.51 | *hsp90aa1* | 1.04 | *ccnc* | -0.43 | *dnaja3* | -0.84 | *annexin* | -1.06 |
| *hspa8* | -0.49 | *por* | 1.05 | *faslg* | -0.42 | *homx1* | -0.80 | *st13* | -1.01 |
| *tnfsf10* | -0.43 | *bax* | 1.18 | *annexin* | -0.38 | *bax* | -0.74 | *ptgs1* | -0.98 |
| *ccnd1* | -0.42 | *hspb1* | 1.19 | *sod* | -0.38 | *hspd1* | -0.74 | *hspa5* | -0.89 |
| *ccng1* | -0.40 | *ddit3* | 1.34 | *gstm3* | -0.35 | *casp8* | -0.72 | *hspa14* | -0.71 |
| *p53* | -0.40 | *p53* | 1.35 | *cat* | -0.23 | *pcna* | -0.70 | *serpine2* | -0.56 |
| *hspd1* | -0.38 | *mt2* | 1.39 | *fmo5* | -0.22 | *ercc3* | -0.55 | *ercc3* | -0.54 |
| *hspa14* | -0.38 | *hspa14* | 1.56 | *vtg1* | -0.20 | *hsp90b1* | -0.54 | *casp8* | -0.53 |
| *ercc1* | -0.33 | *atm* | 1.73 | *cxcr3.1* | -0.09 | *ccng1* | -0.53 | *xrcc2* | -0.42 |
| *por* | -0.32 | *hspa8* | 1.79 | *unga* | -0.06 | *hspe1* | -0.52 | *por* | -0.41 |
| *gsr* | -0.31 | *hspa9* | 1.79 | *mif* | -0.05 | *gstm3* | -0.46 | *hspd1* | -0.40 |
| *hsp90aa1* | -0.28 | *nfkbiab* | 1.83 | *rad50* | -0.03 | *hspa4* | -0.41 | *dnaja3* | -0.31 |
| *annexin* | -0.22 | *hspe1* | 1.89 | *mdm2* | -0.03 | *hspa14* | -0.35 | *hsp70* | -0.30 |
| *faslg* | -0.21 | *casp8* | 1.93 | *casp8* | 0.07 | *ercc1* | -0.33 | *bax* | -0.20 |
| *dnaja3* | -0.05 | *faslg* | 1.99 | *bax* | 0.16 | *serpine2* | -0.31 | *tnfr* | -0.11 |
| *b1p1* | -0.04 | *ccng1* | 2.02 | *tnfsf10* | 0.21 | *faslg* | -0.28 | *ddb1* | 0.07 |
| *casp8* | 0.02 | *rad50* | 2.04 | *ercc3* | 0.24 | *rad23aa* | -0.27 | *gstm3* | 0.07 |
| *egr1* | 0.04 | *mdm2* | 2.33 | *hspd1* | 0.34 | *b1p1* | -0.06 | *hspe1* | 0.12 |
| *nsfa* | 0.05 | *chk2* | 2.38 | *hsp70* | 0.37 | *gsr* | -0.06 | *ddit3* | 0.15 |
| *rad50* | 0.08 | *rad23aa* | 2.41 | *ccnd1* | 0.38 | *nfkbiab* | 0.16 | *gadd45ab* | 0.21 |
| *hspa4* | 0.09 | *tnfsf10* | 2.53 | *ddb1* | 0.43 | *annexin* | 0.18 | *nsfa* | 0.30 |
| *gstm3* | 0.12 | *dnaja3* | 2.61 | *cdkn1a* | 0.46 | *ddit3* | 0.37 | *faslg* | 0.30 |
| *ptgs1* | 0.14 | *nsfa* | 2.66 | *nfkbiab* | 0.56 | *ccnc* | 0.38 | *hspa9* | 0.39 |
| *st13* | 0.17 | *e2f1* | 2.66 | gadd45ab | 0.58 | *por* | 0.44 | *traf4a* | 0.64 |
| *unga* | 0.19 | *gstm3* | 2.67 | egr1 | 0.59 | *chk2* | 0.48 | *mif* | 0.84 |
| *chk2* | 0.20 | *st13* | 2.67 | dnaja3 | 0.63 | *tnfr* | 0.63 | *ccnc* | 0.91 |
| *ddb1* | 0.21 | *hspa4* | 2.71 | pcna | 0.65 | *mif* | 0.85 | *pcna* | 1.02 |
| *pcna* | 0.48 | *b1p1* | 2.79 | homx1 | 0.68 | *hspa8* | 0.85 | *hspb1* | 1.33 |
| *ccnc* | 0.64 | *pcna* | 2.94 | ercc1 | 0.93 | *hsp70* | 0.93 | *nfkbiab* | 1.35 |
| *rad23aa* | 0.66 | *ptgs1* | 3.04 | hspa14 | 0.96 | *cdkn1a* | 1.75 | *prdx2* | 1.36 |
| *traf4a* | 0.75 | *homx1* | 3.04 | st13 | 1.10 | *tnfsf10* | 2.09 | *tnfsf10* | 1.44 |
| *cdkn1a* | 0.77 | *ddb1* | 3.09 | b1p1 | 1.37 | *p53* | 2.12 | *hspa8* | 1.71 |
| *atm* | 0.78 | *sod* | 3.17 | nsfa | 1.41 | *xrcc2* | 2.35 | *ercc1* | 1.96 |
| *homx1* | 0.78 | *ccnc* | 3.14 | hspa4 | 1.54 | *sod* | 2.49 | *homx1* | 2.10 |
| *sod* | 0.79 | *ercc1* | 3.24 | hspa9 | 1.60 | *utg1ab* | 2.75 | *chk2* | 2.11 |
| *nfkbiab* | 0.84 | *unga* | 3.28 | hspb1 | 1.69 | *prdx2* | 2.76 | *sod* | 2.69 |
| *mif* | 0.88 | *cxcr3.1* | 3.29 | atm | 1.82 | *atm* | 2.81 | *utg1ab* | 3.01 |
| *cyp2y3* | 0.92 | *utg1ab* | 3.41 | rad23aa | 2.39 | *cxcr3.1* | 3.33 | *mt2* | 3.20 |
| *ercc3* | 0.93 | *ercc3* | 3.44 | gsr | 2.42 | *mt2* | 3.58 | *adh8a* | 3.21 |
| *mt2* | 1.06 | *traf4a* | 3.52 | p53 | 2.55 | *adh8a* | 3.95 | *unga* | 3.70 |
| *xrcc2* | 1.11 | *mif* | 4.01 | traf4a | 2.59 | *unga* | 4.24 | *cdkn1a* | 3.81 |
| *utg1ab* | 1.87 | *xrcc2* | 4.29 | tnfr | 2.91 | *cyp2y3* | 4.98 | *cyp2y3* | 3.90 |
| *adh8a* | 2.21 | *cyp2y3* | 5.13 | por | 3.32 | *hspa5* | 5.91 | *gsr* | 4.24 |
| *prdx2* | 2.30 | *prdx2* | 5.41 | ddit3 | 3.41 | *ptgs1* | 6.55 | *hsp90b1* | 4.63 |
| *cat* | 2.35 | *cat* | 6.34 | hsp90b1 | 3.56 | *fmo5* | 6.72 | *fmo5* | 5.80 |
| *hsp70* | 3.12 | *cyp1a1* | 6.57 | ptgs1 | 5.78 | *cyp1a1* | 7.70 | *atm* | 6.67 |
| *cyp1a1* | 9.56 | *adh8a* | 7.07 | hspa5 | 6.24 | *hsp90aa1* | 10.80 | *cyp1a1* | 7.53 |
| *vtg1* | 13.34 | *vtg1* | 10.64 | hsp90aa1 | 10.96 | *vtg1* | 19.78 | *vtg1* | 15.72 |

**Table S3 (*Continued***)

| E2_low  and TCDD | | arsenic  and lindane | | arsenic  and TCDD | | lindane  and TCDD | | E2_low  and lindane | |
| --- | --- | --- | --- | --- | --- | --- | --- | --- | --- |
| Genes | MRDs | Genes | MRDs | Genes | MRDs | Genes | MRDs | Genes | MRDs |
| *xrcc2* | -1.14 | *cxcr3.1* | -1.74 | *e2f1* | -2.06 | *hsp90aa1* | -2.23 | *cxcr3.1* | -1.29 |
| *fmo5* | -0.94 | *faslg* | -1.58 | *xrcc2* | -1.87 | *fmo5* | -1.65 | *annexin* | -0.89 |
| *serpine2* | -0.90 | *nfkbiab* | -1.47 | *faslg* | -1.47 | *gadd45ab* | -1.32 | *fmo5* | -0.56 |
| *e2f1* | -0.77 | *xrcc2* | -1.39 | *mdm2* | -1.36 | *cdkn1a* | -0.47 | *p53* | -0.50 |
| *homx1* | -0.64 | *atm* | -1.12 | *pcna* | -1.33 | *nfkbiab* | -0.46 | *serpine2* | 0.30 |
| *hspe1* | -0.55 | *cdkn1a* | -1.08 | *cat* | -1.27 | *xrcc2* | -0.41 | *mt2* | 1.01 |
| *cxcr3.1* | -0.46 | *gstm3* | -0.98 | *ercc3* | -1.18 | *hspa5* | -0.40 | *xrcc2* | 1.34 |
| *tnfsf10* | -0.41 | *prdx2* | -0.93 | *st13* | -1.10 | *atm* | -0.25 | *gadd45ab* | 1.36 |
| *mt2* | -0.35 | *annexin* | -0.93 | *gadd45ab* | -1.09 | *egr1* | -0.19 | *tnfsf10* | 1.89 |
| *unga* | -0.31 | *adh8a* | -0.86 | *bax* | -1.04 | *e2f1* | -0.13 | *ptgs1* | 1.96 |
| *ccng1* | -0.26 | *sod* | -0.85 | *hspb1* | -0.91 | *chk2* | -0.09 | *hsp70* | 1.96 |
| *hspa8* | -0.25 | *utg1ab* | -0.71 | *nfkbiab* | -0.87 | *gsr* | -0.06 | *ccnd1* | 2.40 |
| *annexin* | -0.25 | *gadd45ab* | -0.41 | *ccnd1* | -0.81 | *hspd1* | -0.05 | *bax* | 2.40 |
| *cyp2y3* | -0.16 | *serpine2* | -0.39 | *serpine2* | -0.80 | *faslg* | -0.04 | *ccng1* | 2.50 |
| *mdm2* | 0.03 | *tnfsf10* | -0.37 | *chk2* | -0.80 | *serpine2* | -0.03 | *hspa8* | 2.65 |
| *bax* | 0.04 | *p53* | -0.36 | *hspa9* | -0.79 | *pcna* | 0.18 | *faslg* | 2.66 |
| *faslg* | 0.28 | *e2f1* | -0.36 | *ercc1* | -0.76 | *unga* | 0.33 | *hspe1* | 2.71 |
| *gadd45ab* | 0.56 | *casp8* | -0.34 | *nsfa* | -0.74 | *p53* | 0.69 | *hspd1* | 2.72 |
| *ccnd1* | 0.56 | *cat* | -0.30 | *casp8* | -0.71 | *bax* | 0.72 | *unga* | 2.74 |
| *mif* | 0.87 | *bax* | -0.27 | *dnaja3* | -0.53 | *annexin* | 0.82 | *cdkn1a* | 2.76 |
| *prdx2* | 0.90 | *unga* | -0.23 | *ddb1* | -0.44 | *hsp90b1* | 0.86 | *e2f1* | 2.89 |
| *sod* | 0.99 | *ccnd1* | -0.20 | *hsp90b1* | -0.34 | *utg1ab* | 0.93 | *nfkbiab* | 3.01 |
| *hspb1* | 1.12 | *cyp2y3* | -0.16 | *mif* | -0.26 | *tnfr* | 0.96 | *casp8* | 3.34 |
| *gstm3* | 1.16 | *gsr* | -0.16 | *rad50* | -0.25 | *por* | 1.11 | *sod* | 3.37 |
| *ccnc* | 1.18 | *hspa8* | -0.10 | *ccng1* | -0.25 | *ccnd1* | 1.19 | *mdm2* | 3.41 |
| *hspa14* | 1.34 | *cyp1a1* | 0.06 | *ccnc* | -0.23 | *rad50* | 1.19 | *utg1ab* | 3.67 |
| *casp8* | 1.42 | *pcna* | 0.34 | *hspa14* | -0.12 | *casp8* | 1.44 | *gstm3* | 3.70 |
| *adh8a* | 1.47 | *ccnc* | 0.39 | *gstm3* | -0.12 | *st13* | 1.45 | *ccnc* | 3.75 |
| *dnaja3* | 1.50 | *mt2* | 0.39 | *hspa4* | -0.11 | *rad23aa* | 1.49 | *mif* | 4.00 |
| *ercc3* | 1.71 | *st13* | 0.67 | *traf4a* | -0.11 | *mdm2* | 1.50 | *prdx2* | 4.01 |
| *ddb1* | 1.73 | *chk2* | 0.70 | *hspe1* | -0.08 | *nsfa* | 1.54 | *chk2* | 4.01 |
| *ercc1* | 1.80 | *rad23aa* | 0.70 | *gsr* | -0.05 | *ddit3* | 1.72 | *cyp2y3* | 4.05 |
| *chk2* | 1.82 | *nsfa* | 0.82 | *cdkn1a* | -0.03 | *sod* | 1.75 | *hspa14* | 4.07 |
| *p53* | 1.89 | *mdm2* | 0.95 | *ddit3* | 0.00 | *tnfsf10* | 1.75 | *gsr* | 4.20 |
| *nfkbiab* | 2.03 | *ddb1* | 0.97 | *prdx2* | 0.11 | *b1p1* | 1.79 | *ercc3* | 4.23 |
| *utgbiab* | 2.12 | *mif* | 1.03 | *rad23aa* | 0.11 | *hsp70* | 1.87 | *ddb1* | 4.61 |
| *hspa9* | 2.33 | *hspe1* | 1.08 | *utg1ab* | 0.27 | *vtg1* | 1.91 | *egr1* | 4.61 |
| *st13* | 2.39 | *hspd1* | 1.11 | *annexin* | 0.37 | *cyp1a1* | 1.92 | *dnaja3* | 4.76 |
| *b1p1* | 2.65 | *egr1* | 1.20 | *atm* | 0.79 | *ercc1* | 1.93 | *st13* | 4.89 |
| *hspa4* | 2.71 | *ercc3* | 1.25 | *sod* | 1.07 | *gstm3* | 1.96 | *tnfr* | 5.18 |
| *nsfa* | 2.80 | *hspb1* | 1.46 | *cyp1a1* | 1.08 | *hspa4* | 1.99 | *pcna* | 5.31 |
| *cdkn1a* | 2.83 | *traf4a* | 1.50 | *b1p1* | 1.09 | *ercc3* | 2.05 | *hspa4* | 5.33 |
| *pcna* | 2.84 | *hspa4* | 1.56 | *por* | 1.09 | *ptgs1* | 2.06 | *hspb1* | 5.39 |
| *ddit3* | 3.04 | *rad50* | 1.66 | *adh8a* | 1.12 | *ccnc* | 2.16 | *nsfa* | 5.40 |
| *hspd1* | 3.13 | *ercc1* | 2.04 | *tnfr* | 1.19 | *hspa14* | 2.18 | *b1p1* | 5.49 |
| *tnfr* | 3.47 | *hspa9* | 2.09 | *unga* | 1.30 | *ddb1* | 2.24 | *por* | 5.59 |
| *gsr* | 3.71 | *hsp90b1* | 2.10 | *tnfsf10* | 1.31 | *hspa8* | 2.27 | *atm* | 5.59 |
| *rad23aa* | 3.85 | *homx1* | 2.11 | *hspa8* | 1.34 | *traf4a* | 2.32 | *hspa9* | 5.59 |
| *cat* | 3.88 | *dnaja3* | 2.49 | *egr1* | 1.36 | *hspe1* | 2.50 | *rad23aa* | 5.60 |
| *por* | 3.96 | *hsp70* | 2.52 | *p53* | 1.46 | *cxcr3.1* | 2.65 | *ercc1* | 5.64 |
| *rad50* | 4.14 | *ccng1* | 2.57 | *hspd1* | 1.53 | *hspa9* | 2.67 | *ddit3* | 6.00 |
| *atm* | 4.64 | *hspa14* | 2.58 | *homx1* | 2.20 | *hspb1* | 2.68 | *homx1* | 6.01 |
| *traf4a* | 4.65 | *por* | 2.71 | *cxcr3.1* | 2.69 | *mif* | 2.68 | *rad50* | 6.10 |
| *egr1* | 4.77 | *ptgs1* | 2.73 | *cyp2y3* | 3.53 | *ccng1* | 2.70 | *hsp90aa1* | 6.24 |
| *ptgs1* | 4.81 | *fmo5* | 2.77 | *vtg1* | 4.45 | *prdx2* | 2.76 | *adh8a* | 6.32 |
| *hsp70* | 4.90 | *tnfr* | 2.90 | *fmo5* | 4.46 | *cat* | 3.09 | *cyp1a1* | 6.41 |
| *hsp90b1* | 4.94 | *ddit3* | 2.96 | *hsp90aa1* | 4.83 | *dnaja3* | 3.16 | *vtg1* | 6.58 |
| *hsp90aa1* | 4.99 | *b1p1* | 3.92 | *hspa5* | 5.39 | *cyp2y3* | 3.68 | *hspa5* | 6.59 |
| *hspa5* | 5.72 | *hsp90aa1* | 6.08 | *hsp70* | 5.47 | *adh8a* | 4.23 | *cat* | 7.05 |
| *vtg1* | 9.32 | *hspa5* | 6.26 | *ptgs1* | 5.58 | *mt2* | 4.40 | *traf4a* | 7.42 |
| *cyp1a1* | 9.40 | *vtg1* | 8.32 | *mt2* | 6.59 | *homx1* | 6.11 | *hsp90b1* | 7.50 |

^a^TCDD: 5 nM 2,3,7,8-tetrachlorodibenzo-*p*-dioxin; lindane: 100 µg/L lindane; arsenic: 15 mg/L arsenic; E2_low: 5 µg/L 17β-estradiol; E2_high: 50 µg/L 17β-estradiol; ^b^Genes that can be used to differentiate the corresponding pair of chemicals were highlighted in red; ^c^Full names of genes can be found in Supporting Information, Table S1 or S2.
