# Supplementary material for: Selection of Reliable Biomarkers from PCR Array Analyses Using Relative Distance Computational Model: Methodology and Proof-of-Concept Study
Source: PLoS One. 2013 Dec 12;8(12):e83954. doi: 10.1371/journal.pone.0083954 (PMC3861511; doi:10.1371/journal.pone.0083954)
Supplement: Table S4 — Maximum mean relative distances (MMRDs) of gene sets with different amounts of genes among 5 chemicals/concentrations and the corresponding components of genes. (DOC) [file pone.0083954.s004.doc]

**Table S4 Maximum mean relative distances (MMRDs) of gene sets with different amounts of genes among 5 chemicals/concentrations and the corresponding components of genes^a,b^**.

| Number of genes | MMRDs | *Components of genes* |
| --- | --- | --- |
| 1 | 8.991 | *vtg1* |
| 2 | 10.568 | *vtg1, hspa5* |
| 3 | 12.08 | *vtg1, hspa5, cyp1a1* |
| 4 | 12.81 | *vtg1, hspa5, cyp1a1, ptgs1* |
| 5 | 13.59 | *vtg1, hspa5, cyp1a1, homx1, hsp90aa1* |
| 6 | 14.18 | *vtg1, hspa5, cyp1a1, homx1, hsp90aa1, ptgs1* |
| 7 | 14.58 | *vtg1, hspa5, cyp1a1, homx1, hsp90aa1, ptgs1, adh8a* |
| 8 | 14.93 | *vtg1, hspa5, cyp1a1, homx1, hsp90aa1, ptgs1, adh8a, cyp2y3* |
| 9 | 15.27 | *vtg1, hspa5, cyp1a1, homx1, hsp90aa1, ptgs1, adh8a, cyp2y3, mt2* |
| 10 | 15.56 | *vtg1, hspa5, cyp1a1, homx1, hsp90aa1, ptgs1, adh8a, cyp2y3, mt2, hsp70* |
| 11 | 15.85 | *vtg1, hspa5, cyp1a1, homx1, hsp90aa1, ptgs1, adh8a, cyp2y3, mt2, hsp70, hsp90b1* |
| 12 | 16.11 | *vtg1, hspa5, cyp1a1, homx1, hsp90aa1, ptgs1, adh8a, cyp2y3, mt2, hsp70, hsp90b1, atm* |
| 13 | 16.30 | *vtg1, hspa5, cyp1a1, homx1, hsp90aa1, ptgs1, adh8a, cyp2y3, mt2, hsp70, hsp90b1, atm, ddit3* |
| 14 | 16.47 | *vtg1, hspa5, cyp1a1, homx1, hsp90aa1, ptgs1, adh8a, cyp2y3, mt2, hsp70, hsp90b1, atm, ddit3, traf4a* |
| 15 | 16.64 | *vtg1, hspa5, cyp1a1, homx1, hsp90aa1, ptgs1, adh8a, cyp2y3, mt2, hsp70, hsp90b1, atm, ddit3, traf4a, b1p1* |
| 16 | 16.79 | *vtg1, hspa5, cyp1a1, homx1, hsp90aa1, ptgs1, adh8a, cyp2y3, mt2, hsp70, hsp90b1, atm, ddit3, traf4a, b1p1, tnfr* |
| 17 | 16.95 | *vtg1, hspa5, cyp1a1, homx1, hsp90aa1, ptgs1, adh8a, cyp2y3, mt2, hsp70, hsp90b1, atm, ddit3, traf4a, b1p1, tnfr, por* |
| 18 | 17.12 | *vtg1, hspa5, cyp1a1, homx1, hsp90aa1, ptgs1, adh8a, cyp2y3, mt2, hsp70, hsp90b1, atm, ddit3, traf4a, b1p1, tnfr, por, fmo5* |
| 19 | 17.27 | *vtg1, hspa5, cyp1a1, homx1, hsp90aa1, ptgs1, adh8a, cyp2y3, mt2, hsp70, hsp90b1, atm, ddit3, traf4a, b1p1, tnfr, por, fmo5, ercc1* |
| 20 | 17.39 | *vtg1, hspa5, cyp1a1, homx1, hsp90aa1, ptgs1, adh8a, cyp2y3, mt2, hsp70, hsp90b1, atm, ddit3, traf4a, b1p1, tnfr, por, fmo5, ercc1, dnaja3* |
| 21 | 17.52 | *vtg1, hspa5, cyp1a1, homx1, hsp90aa1, ptgs1, adh8a, cyp2y3, mt2, hsp70, hsp90b1, atm, ddit3, traf4a, b1p1, tnfr, por, fmo5, ercc1, dnaja3, gsr* |
| 22 | 17.65 | *vtg1, hspa5, cyp1a1, homx1, hsp90aa1, ptgs1, adh8a, cyp2y3, mt2, hsp70, hsp90b1, atm, ddit3, traf4a, b1p1, tnfr, por, fmo5, ercc1, dnaja3, gsr, hspa9* |
| 23 | 17.77 | *vtg1, hspa5, cyp1a1, homx1, hsp90aa1, ptgs1, adh8a, cyp2y3, mt2, hsp70, hsp90b1, atm, ddit3, traf4a, b1p1, tnfr, por, fmo5, ercc1, dnaja3, gsr, hspa9, prdx2* |
| 24 | 17.88 | *vtg1, hspa5, cyp1a1, homx1, hsp90aa1, ptgs1, adh8a, cyp2y3, mt2, hsp70, hsp90b1, atm, ddit3, traf4a, b1p1, tnfr, por, fmo5, ercc1, dnaja3, gsr, hspa9, prdx2, sod* |
| 25 | 17.98 | *vtg1, hspa5, cyp1a1, homx1, hsp90aa1, ptgs1, adh8a, cyp2y3, mt2, hsp70, hsp90b1, atm, ddit3, traf4a, b1p1, tnfr, por, fmo5, ercc1, dnaja3, gsr, hspa9, prdx2, sod, unga* |
| 26 | 18.07 | *vtg1, hspa5, cyp1a1, homx1, hsp90aa1, ptgs1, adh8a, cyp2y3, mt2, hsp70, hsp90b1, atm, ddit3, traf4a, b1p1, tnfr, por, fmo5, ercc1, dnaja3, gsr, hspa9, prdx2, sod, unga, utg1ab* |
| 27 | 18.16 | *vtg1, hspa5, cyp1a1, homx1, hsp90aa1, ptgs1, adh8a, cyp2y3, mt2, hsp70, hsp90b1, atm, ddit3, traf4a, b1p1, tnfr, por, fmo5, ercc1, dnaja3, gsr, hspa9, prdx2, sod, unga, utg1ab, nsfa* |
| 28 | 18.24 | *vtg1, hspa5, cyp1a1, homx1, hsp90aa1, ptgs1, adh8a, cyp2y3, mt2, hsp70, hsp90b1, atm, ddit3, traf4a, b1p1, tnfr, por, fmo5, ercc1, dnaja3, gsr, hspa9, prdx2, sod, unga, utg1ab, nsfa, cxcr3.1* |
| 29 | 18.33 | *vtg1, hspa5, cyp1a1, homx1, hsp90aa1, ptgs1, adh8a, cyp2y3, mt2, hsp70, hsp90b1, atm, ddit3, traf4a, b1p1, tnfr, por, fmo5, ercc1, dnaja3, gsr, hspa9, prdx2, sod, unga, utg1ab, nsfa, cxcr3.1, ercc3* |
| 30 | 18.40 | *vtg1, hspa5, cyp1a1, homx1, hsp90aa1, ptgs1, adh8a, cyp2y3, mt2, hsp70, hsp90b1, atm, ddit3, traf4a, b1p1, tnfr, por, fmo5, ercc1, dnaja3, gsr, hspa9, prdx2, sod, unga, utg1ab, nsfa, cxcr3.1, ercc3, ccnc* |
| 31 | 18.46 | *vtg1, hspa5, cyp1a1, homx1, hsp90aa1, ptgs1, adh8a, cyp2y3, mt2, hsp70, hsp90b1, atm, ddit3, traf4a, b1p1, tnfr, por, fmo5, ercc1, dnaja3, gsr, hspa9, prdx2, sod, unga, utg1ab, nsfa, cxcr3.1, ercc3, ccnc, hspb1* |
| 32 | 18.51 | *vtg1, hspa5, cyp1a1, homx1, hsp90aa1, ptgs1, adh8a, cyp2y3, mt2, hsp70, hsp90b1, atm, ddit3, traf4a, b1p1, tnfr, por, fmo5, ercc1, dnaja3, gsr, hspa9, prdx2, sod, unga, utg1ab, nsfa, cxcr3.1, ercc3, ccnc, hspb1, pcna* |
| 33 | 18.57 | *vtg1, hspa5, cyp1a1, homx1, hsp90aa1, ptgs1, adh8a, cyp2y3, mt2, hsp70, hsp90b1, atm, ddit3, traf4a, b1p1, tnfr, por, fmo5, ercc1, dnaja3, gsr, hspa9, prdx2, sod, unga, utg1ab, nsfa, cxcr3.1, ercc3, ccnc, hspb1, pcna, egr1* |
| 34 | 18.63 | *vtg1, hspa5, cyp1a1, homx1, hsp90aa1, ptgs1, adh8a, cyp2y3, mt2, hsp70, hsp90b1, atm, ddit3, traf4a, b1p1, tnfr, por, fmo5, ercc1, dnaja3, gsr, hspa9, prdx2, sod, unga, utg1ab, nsfa, cxcr3.1, ercc3, ccnc, hspb1, pcna, egr1, ddb1* |
| 35 | 18.69 | *vtg1, hspa5, cyp1a1, homx1, hsp90aa1, ptgs1, adh8a, cyp2y3, mt2, hsp70, hsp90b1, atm, ddit3, traf4a, b1p1, tnfr, por, fmo5, ercc1, dnaja3, gsr, hspa9, prdx2, sod, unga, utg1ab, nsfa, cxcr3.1, ercc3, ccnc, hspb1, pcna, egr1, ddb1, mif* |
| 36 | 18.75 | *vtg1, hspa5, cyp1a1, homx1, hsp90aa1, ptgs1, adh8a, cyp2y3, mt2, hsp70, hsp90b1, atm, ddit3, traf4a, b1p1, tnfr, por, fmo5, ercc1, dnaja3, gsr, hspa9, prdx2, sod, unga, utg1ab, nsfa, cxcr3.1, ercc3, ccnc, hspb1, pcna, egr1, ddb1, mif, hspa14* |
| 37 | 18.80 | *vtg1, hspa5, cyp1a1, homx1, hsp90aa1, ptgs1, adh8a, cyp2y3, mt2, hsp70, hsp90b1, atm, ddit3, traf4a, b1p1, tnfr, por, fmo5, ercc1, dnaja3, gsr, hspa9, prdx2, sod, unga, utg1ab, nsfa, cxcr3.1, ercc3, ccnc, hspb1, pcna, egr1, ddb1, mif, hspa14, p53* |
| 38 | 18.85 | *vtg1, hspa5, cyp1a1, homx1, hsp90aa1, ptgs1, adh8a, cyp2y3, mt2, hsp70, hsp90b1, atm, ddit3, traf4a, b1p1, tnfr, por, fmo5, ercc1, dnaja3, gsr, hspa9, prdx2, sod, unga, utg1ab, nsfa, cxcr3.1, ercc3, ccnc, hspb1, pcna, egr1, ddb1, mif, hspa14, p53, hspa4* |

**Table S4 (*Continued a***)

| Number of genes | MMRDs | *Components of genes* |
| --- | --- | --- |
| 39 | 18.90 | *vtg1, hspa5, cyp1a1, homx1, hsp90aa1, ptgs1, adh8a, cyp2y3, mt2, hsp70, hsp90b1, atm, ddit3, traf4a, b1p1, tnfr, por, fmo5, ercc1, dnaja3, gsr, hspa9, prdx2, sod, unga, utg1ab, nsfa, cxcr3.1, ercc3, ccnc, hspb1, pcna, egr1, ddb1, mif, hspa14, p53, hspa4, gstm3* |
| 40 | 18.94 | *vtg1, hspa5, cyp1a1, homx1, hsp90aa1, ptgs1, adh8a, cyp2y3, mt2, hsp70, hsp90b1, atm, ddit3, traf4a, b1p1, tnfr, por, fmo5, ercc1, dnaja3, gsr, hspa9, prdx2, sod, unga, utg1ab, nsfa, cxcr3.1, ercc3, ccnc, hspb1, pcna, egr1, ddb1, mif, hspa14, p53, hspa4, gstm3, st13* |
| 41 | 18.98 | *vtg1, hspa5, cyp1a1, homx1, hsp90aa1, ptgs1, adh8a, cyp2y3, mt2, hsp70, hsp90b1, atm, ddit3, traf4a, b1p1, tnfr, por, fmo5, ercc1, dnaja3, gsr, hspa9, prdx2, sod, unga, utg1ab, nsfa, cxcr3.1, ercc3, ccnc, hspb1, pcna, egr1, ddb1, mif, hspa14, p53, hspa4, gstm3, st13, hspa8* |
| 42 | 19.02 | *vtg1, hspa5, cyp1a1, homx1, hsp90aa1, ptgs1, adh8a, cyp2y3, mt2, hsp70, hsp90b1, atm, ddit3, traf4a, b1p1, tnfr, por, fmo5, ercc1, dnaja3, gsr, hspa9, prdx2, sod, unga, utg1ab, nsfa, cxcr3.1, ercc3, ccnc, hspb1, pcna, egr1, ddb1, mif, hspa14, p53, hspa4, gstm3, st13, hspa8, cdkn1a* |
| 43 | 19.05 | *vtg1, hspa5, cyp1a1, homx1, hsp90aa1, ptgs1, adh8a, cyp2y3, mt2, hsp70, hsp90b1, atm, ddit3, traf4a, b1p1, tnfr, por, fmo5, ercc1, dnaja3, gsr, hspa9, prdx2, sod, unga, utg1ab, nsfa, cxcr3.1, ercc3, ccnc, hspb1, pcna, egr1, ddb1, mif, hspa14, p53, hspa4, gstm3, st13, hspa8, cdkn1a, rad23aa* |
| 44 | 19.07 | *vtg1, hspa5, cyp1a1, homx1, hsp90aa1, ptgs1, adh8a, cyp2y3, mt2, hsp70, hsp90b1, atm, ddit3, traf4a, b1p1, tnfr, por, fmo5, ercc1, dnaja3, gsr, hspa9, prdx2, sod, unga, utg1ab, nsfa, cxcr3.1, ercc3, ccnc, hspb1, pcna, egr1, ddb1, mif, hspa14, p53, hspa4, gstm3, st13, hspa8, cdkn1a, rad23aa, rad50* |
| 45 | 19.09 | *vtg1, hspa5, cyp1a1, homx1, hsp90aa1, ptgs1, adh8a, cyp2y3, mt2, hsp70, hsp90b1, atm, ddit3, traf4a, b1p1, tnfr, por, fmo5, ercc1, dnaja3, gsr, hspa9, prdx2, sod, unga, utg1ab, nsfa, cxcr3.1, ercc3, ccnc, hspb1, pcna, egr1, ddb1, mif, hspa14, p53, hspa4, gstm3, st13, hspa8, cdkn1a, rad23aa, rad50, chk2* |
| 46 | 19.12 | *vtg1, hspa5, cyp1a1, homx1, hsp90aa1, ptgs1, adh8a, cyp2y3, mt2, hsp70, hsp90b1, atm, ddit3, traf4a, b1p1, tnfr, por, fmo5, ercc1, dnaja3, gsr, hspa9, prdx2, sod, unga, utg1ab, nsfa, cxcr3.1, ercc3, ccnc, hspb1, pcna, egr1, ddb1, mif, hspa14, p53, hspa4, gstm3, st13, hspa8, cdkn1a, rad23aa, rad50, chk2, hspe1* |
| 47 | 19.14 | *vtg1, hspa5, cyp1a1, homx1, hsp90aa1, ptgs1, adh8a, cyp2y3, mt2, hsp70, hsp90b1, atm, ddit3, traf4a, b1p1, tnfr, por, fmo5, ercc1, dnaja3, gsr, hspa9, prdx2, sod, unga, utg1ab, nsfa, cxcr3.1, ercc3, ccnc, hspb1, pcna, egr1, ddb1, mif, hspa14, p53, hspa4, gstm3, st13, hspa8, cdkn1a, rad23aa, rad50, chk2, hspe1, hspd1* |
| 48 | 19.146 | *vtg1, hspa5, cyp1a1, homx1, hsp90aa1, ptgs1, adh8a, cyp2y3, mt2, hsp70, hsp90b1, atm, ddit3, traf4a, b1p1, tnfr, por, fmo5, ercc1, dnaja3, gsr, hspa9, prdx2, sod, unga, utg1ab, nsfa, cxcr3.1, ercc3, ccnc, hspb1, pcna, egr1, ddb1, mif, hspa14, p53, hspa4, gstm3, st13, hspa8, cdkn1a, rad23aa, rad50, chk2, hspe1, hspd1, annexin* |
| 49 | 19.148 | *vtg1, hspa5, cyp1a1, homx1, hsp90aa1, ptgs1, adh8a, cyp2y3, mt2, hsp70, hsp90b1, atm, ddit3, traf4a, b1p1, tnfr, por, fmo5, ercc1, dnaja3, gsr, hspa9, prdx2, sod, unga, utg1ab, nsfa, cxcr3.1, ercc3, ccnc, hspb1, pcna, egr1, ddb1, mif, hspa14, p53, hspa4, gstm3, st13, hspa8, cdkn1a, rad23aa, rad50, chk2, hspe1, hspd1, annexin, bax* |
| 50 | 19.153 | *vtg1, hspa5, cyp1a1, homx1, hsp90aa1, ptgs1, adh8a, cyp2y3, mt2, hsp70, hsp90b1, atm, ddit3, traf4a, b1p1, tnfr, por, fmo5, ercc1, dnaja3, gsr, hspa9, prdx2, sod, unga, utg1ab, nsfa, cxcr3.1, ercc3, ccnc, hspb1, pcna, egr1, ddb1, mif, hspa14, p53, hspa4, gstm3, st13, hspa8, cdkn1a, rad23aa, rad50, chk2, hspe1, hspd1, annexin, bax, tnfsf10* |
| 51 | 19.151 | *vtg1, hspa5, cyp1a1, homx1, hsp90aa1, ptgs1, adh8a, cyp2y3, mt2, hsp70, hsp90b1, atm, ddit3, traf4a, b1p1, tnfr, por, fmo5, ercc1, dnaja3, gsr, hspa9, prdx2, sod, unga, utg1ab, nsfa, cxcr3.1, ercc3, ccnc, hspb1, pcna, egr1, ddb1, mif, hspa14, p53, hspa4, gstm3, st13, hspa8, cdkn1a, rad23aa, rad50, chk2, hspe1, hspd1, annexin, bax, tnfsf10, casp8* |
| 52 | 19.14 | *vtg1, hspa5, cyp1a1, homx1, hsp90aa1, ptgs1, adh8a, cyp2y3, mt2, hsp70, hsp90b1, atm, ddit3, traf4a, b1p1, tnfr, por, fmo5, ercc1, dnaja3, gsr, hspa9, prdx2, sod, unga, utg1ab, nsfa, cxcr3.1, ercc3, ccnc, hspb1, pcna, egr1, ddb1, mif, hspa14, p53, hspa4, gstm3, st13, hspa8, cdkn1a, rad23aa, rad50, chk2, hspe1, hspd1, annexin, bax, tnfsf10, casp8, cat* |
| 53 | 19.13 | *vtg1, hspa5, cyp1a1, homx1, hsp90aa1, ptgs1, adh8a, cyp2y3, mt2, hsp70, hsp90b1, atm, ddit3, traf4a, b1p1, tnfr, por, fmo5, ercc1, dnaja3, gsr, hspa9, prdx2, sod, unga, utg1ab, nsfa, cxcr3.1, ercc3, ccnc, hspb1, pcna, egr1, ddb1, mif, hspa14, p53, hspa4, gstm3, st13, hspa8, cdkn1a, rad23aa, rad50, chk2, hspe1, hspd1, annexin, bax, tnfsf10, casp8, cat, ccng1* |
| 54 | 19.10 | *vtg1, hspa5, cyp1a1, homx1, hsp90aa1, ptgs1, adh8a, cyp2y3, mt2, hsp70, hsp90b1, atm, ddit3, traf4a, b1p1, tnfr, por, fmo5, ercc1, dnaja3, gsr, hspa9, prdx2, sod, unga, utg1ab, nsfa, cxcr3.1, ercc3, ccnc, hspb1, pcna, egr1, ddb1, mif, hspa14, p53, hspa4, gstm3, st13, hspa8, cdkn1a, rad23aa, rad50, chk2, hspe1, hspd1, annexin, bax, tnfsf10, casp8, cat, ccng1, mdm2* |
| 55 | 19.06 | *vtg1, hspa5, cyp1a1, homx1, hsp90aa1, ptgs1, adh8a, cyp2y3, mt2, hsp70, hsp90b1, atm, ddit3, traf4a, b1p1, tnfr, por, fmo5, ercc1, dnaja3, gsr, hspa9, prdx2, sod, unga, utg1ab, nsfa, cxcr3.1, ercc3, ccnc, hspb1, pcna, egr1, ddb1, mif, hspa14, p53, hspa4, gstm3, st13, hspa8, cdkn1a, rad23aa, rad50, chk2, hspe1, hspd1, annexin, bax, tnfsf10, casp8, cat, ccng1, mdm2, nfkbiab* |
| 56 | 19.01 | *vtg1, hspa5, cyp1a1, homx1, hsp90aa1, ptgs1, adh8a, cyp2y3, mt2, hsp70, hsp90b1, atm, ddit3, traf4a, b1p1, tnfr, por, fmo5, ercc1, dnaja3, gsr, hspa9, prdx2, sod, unga, utg1ab, nsfa, cxcr3.1, ercc3, ccnc, hspb1, pcna, egr1, ddb1, mif, hspa14, p53, hspa4, gstm3, st13, hspa8, cdkn1a, rad23aa, rad50, chk2, hspe1, hspd1, annexin, bax, tnfsf10, casp8, cat, ccng1, mdm2, nfkbiab, ccnd1* |
| 57 | 18.95 | *vtg1, hspa5, cyp1a1, homx1, hsp90aa1, ptgs1, adh8a, cyp2y3, mt2, hsp70, hsp90b1, atm, ddit3, traf4a, b1p1, tnfr, por, fmo5, ercc1, dnaja3, gsr, hspa9, prdx2, sod, unga, utg1ab, nsfa, cxcr3.1, ercc3, ccnc, hspb1, pcna, egr1, ddb1, mif, hspa14, p53, hspa4, gstm3, st13, hspa8, cdkn1a, rad23aa, rad50, chk2, hspe1, hspd1, annexin, bax, tnfsf10, casp8, cat, ccng1, mdm2, nfkbiab, ccnd1, serpine2* |
| 58 | 18.90 | *vtg1, hspa5, cyp1a1, homx1, hsp90aa1, ptgs1, adh8a, cyp2y3, mt2, hsp70, hsp90b1, atm, ddit3, traf4a, b1p1, tnfr, por, fmo5, ercc1, dnaja3, gsr, hspa9, prdx2, sod, unga, utg1ab, nsfa, cxcr3.1, ercc3, ccnc, hspb1, pcna, egr1, ddb1, mif, hspa14, p53, hspa4, gstm3, st13, hspa8, cdkn1a, rad23aa, rad50, chk2, hspe1, hspd1, annexin, bax, tnfsf10, casp8, cat, ccng1, mdm2, nfkbiab, ccnd1, serpine2, faslg* |

**Table S4 (*Continued b***)

| Number of genes | MMRDs | *Components of genes* |
| --- | --- | --- |
| 59 | 18.81 | *vtg1, hspa5, cyp1a1, homx1, hsp90aa1, ptgs1, adh8a, cyp2y3, mt2, hsp70, hsp90b1, atm, ddit3, traf4a, b1p1, tnfr, por, fmo5, ercc1, dnaja3, gsr, hspa9, prdx2, sod, unga, utg1ab, nsfa, cxcr3.1, ercc3, ccnc, hspb1, pcna, egr1, ddb1, mif, hspa14, p53, hspa4, gstm3, st13, hspa8, cdkn1a, rad23aa, rad50, chk2, hspe1, hspd1, annexin, bax, tnfsf10, casp8, cat, ccng1, mdm2, nfkbiab, ccnd1, serpine2, faslg. xrcc2* |
| 60 | 18.71 | *vtg1, hspa5, cyp1a1, homx1, hsp90aa1, ptgs1, adh8a, cyp2y3, mt2, hsp70, hsp90b1, atm, ddit3, traf4a, b1p1, tnfr, por, fmo5, ercc1, dnaja3, gsr, hspa9, prdx2, sod, unga, utg1ab, nsfa, cxcr3.1, ercc3, ccnc, hspb1, pcna, egr1, ddb1, mif, hspa14, p53, hspa4, gstm3, st13, hspa8, cdkn1a, rad23aa, rad50, chk2, hspe1, hspd1, annexin, bax, tnfsf10, casp8, cat, ccng1, mdm2, nfkbiab, ccnd1, serpine2, faslg. xrcc2, e2f1* |
| 61 | 18.62 | *vtg1, hspa5, cyp1a1, homx1, hsp90aa1, ptgs1, adh8a, cyp2y3, mt2, hsp70, hsp90b1, atm, ddit3, traf4a, b1p1, tnfr, por, fmo5, ercc1, dnaja3, gsr, hspa9, prdx2, sod, unga, utg1ab, nsfa, cxcr3.1, ercc3, ccnc, hspb1, pcna, egr1, ddb1, mif, hspa14, p53, hspa4, gstm3, st13, hspa8, cdkn1a, rad23aa, rad50, chk2, hspe1, hspd1, annexin, bax, tnfsf10, casp8, cat, ccng1, mdm2, nfkbiab, ccnd1, serpine2, faslg. xrcc2, e2f1, gadd45ab* |

^a^Genes that can be used to differentiate the corresponding pair of chemicals were highlighted in red; ^b^Full names of genes can be found in Supporting Information, Table S1 or S2.
